# Expert opinion on diagnosis and management of epilepsy‐associated comorbidities

**DOI:** 10.1002/epi4.12851

**Published:** 2023-11-27

**Authors:** Jukka Peltola, Rainer Surges, Berthold Voges, Tim J. von Oertzen

**Affiliations:** ^1^ Faculty of Medicine and Health Technology Tampere University Tampere Finland; ^2^ Department of Neurology Tampere University Hospital Tampere Finland; ^3^ Department of Epileptology University Hospital Bonn Bonn Germany; ^4^ Department of Neurology, Epilepsy Center Hamburg Protestant Hospital Alsterdorf Hamburg Germany; ^5^ Medical Faculty Johannes Kepler University Linz Austria; ^6^ Department of Neurology 1, Neuromed Campus Kepler University Hospital Linz Austria

**Keywords:** cardiovascular diseases, cognitive dysfunction, depression, epilepsy, sleep–wake disorders, sudden cardiac death

## Abstract

Apart from seizure freedom, the presence of comorbidities related to neurological, cardiovascular, or psychiatric disorders is the largest determinant of a reduced health‐related quality of life in people with epilepsy (PwE). However, comorbidities are often underrecognized and undertreated, and clinical management of comorbid conditions can be challenging. The focus of a comprehensive treatment regimen should maximize seizure control while optimizing clinical management of treatable comorbidities to improve a person's quality of life and overall health. A panel of four European epileptologists with expertise in their respective fields of epilepsy‐related comorbidities combined the latest available scientific evidence with clinical expertise and collaborated to provide consensus practical advice to improve the identification and management of comorbidities in PwE. This review provides a critical evaluation for the diagnosis and management of sleep–wake disorders, cardiovascular diseases, cognitive dysfunction, and depression in PwE. Whenever possible, clinical data have been provided. The PubMed database was the main search source for the literature review. The deleterious pathophysiological processes underlying neurological, cardiovascular, or psychiatric comorbidities in PwE interact with the processes responsible for generating seizures to increase cerebral and physiological dysfunction. This can increase the likelihood of developing drug‐resistant epilepsy; therefore, early identification of comorbidities and intervention is imperative. The practical evidence‐based advice presented in this article may help clinical neurologists and other specialist physicians responsible for the care and management of PwE.

**Abbreviations**
ANT: anterior nucleus of the thalamus
ASM: antiseizure medication
DBS: deep brain stimulation
DRE: drug‐resistant epilepsy
EEG: electroencephalogram
OSA: obstructive sleep apnea
PwE: people with epilepsy
REM: rapid eye movement
SCD: sudden cardiac death
SUDEP: sudden unexpected death in epilepsy
TCS: generalized or focal to bilateral tonic tonic–clonic seizures
VNS: vagus nerve stimulation

**Key points**

- Comorbidities can significantly impact health‐related quality of life, seizure frequency, and severity in people with epilepsy.
- Common comorbidities include sleep–wake disorders, cardiovascular disorders, cognitive impairments, and depression.
- Processes underlying such comorbidities increase cerebral and physiological dysfunction, increasing the likelihood of drug‐resistant epilepsy.
- Early identification of comorbidities and intervention is important to improve seizure frequency and severity and improve quality of life.
- Four European epileptologists collaborated to provide consensus practical evidence‐based advice to optimize treatment management.

## 1 INTRODUCTION

Epilepsy is a spectrum disorder with neurological, cardiovascular, and psychological consequences that affects about 50 million individuals worldwide.^1^

The treatment goal in newly diagnosed epilepsy is to enable seizure freedom with minimal side effects. However, despite the availability of various antiseizure treatment options, about one‐third of people with epilepsy (PwE) are unable to achieve sustained seizure freedom even after two adequate, tolerated, appropriately chosen treatments, and they are classified as having drug‐resistant epilepsy (DRE).^2^, ^3^

**FIGURE 1:**
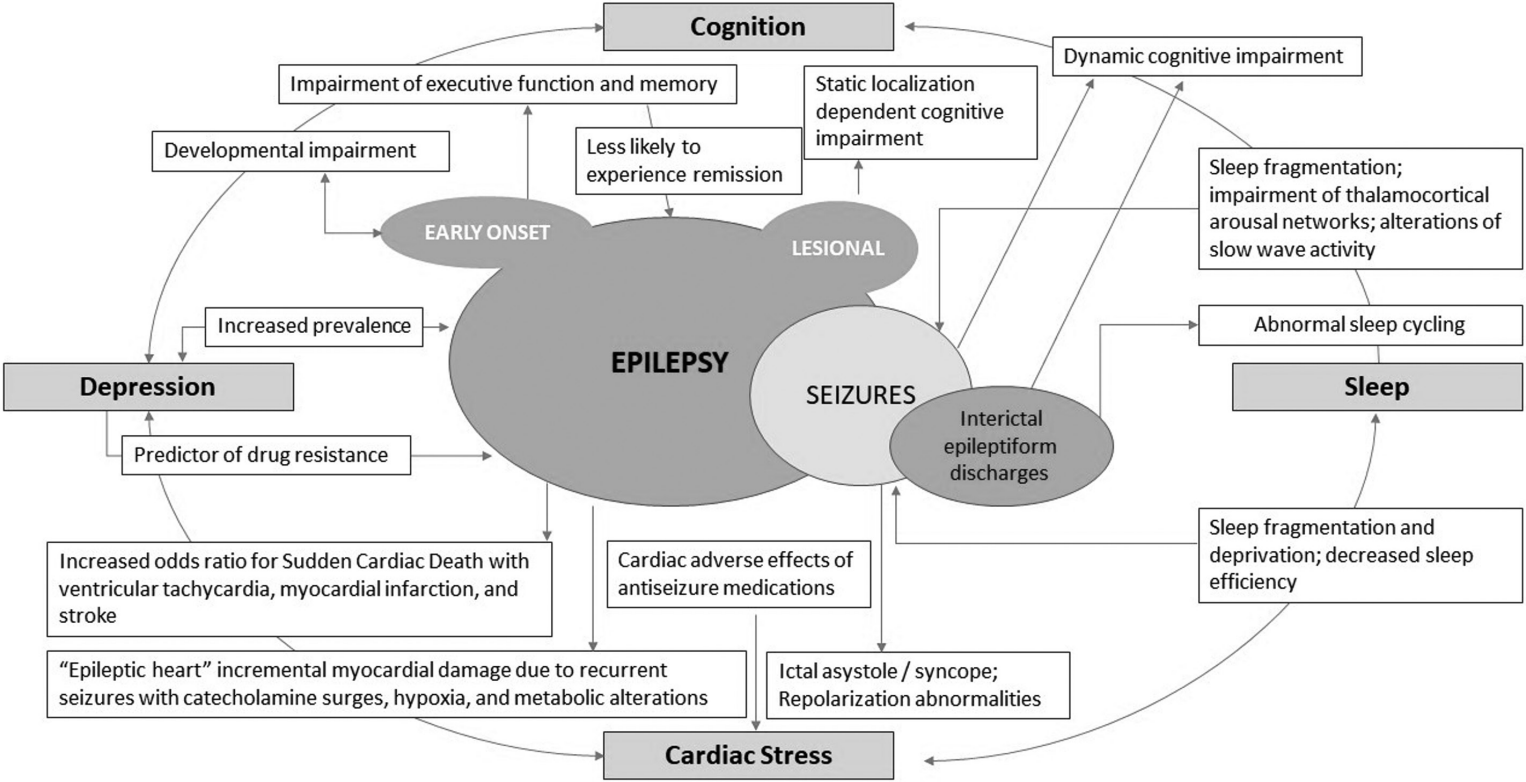
Association between epilepsy and several common comorbidities. Epilepsy has a bidirectional causative association as well as shared risk factors with depression and sleep–wake disorders. The comorbidities of cardiovascular and cognitive dysfunctions are resultant causative effects of epilepsy. An arrow with a solid line represents a casual association with the cause leading to the effect.

Apart from seizure freedom, the presence of comorbidities in PwE is the largest determinant of reduced health‐related quality of life. Although comorbidities lead to a greater disease burden for patients and their caregivers, comorbidities in PwE are often underrecognized and undertreated. The focus of a comprehensive treatment regimen should aim to understand the physiological associations between epilepsy and related comorbidities (Figure 1) with an aim to maximize long‐term seizure control while optimizing the management of associated disorders to improve a person's quality of life and overall health.^4^

Based on our clinical experience as epileptologists and based on the available evidence, we present practical advice for the diagnosis and clinical management of sleep–wake disorders, cardiovascular disorders, cognitive impairments, and depression in PwE.^5^, ^6^, ^7^, ^8^, ^9^, ^10^ We have focused on these comorbidities as they are common in PwE, can significantly impact the quality of life, and may respond to available treatment options.

In Table S1, we have suggested references of interest for additional information.

## 2 EPILEPSY AND SLEEP–WAKE DISORDERS

**FIGURE 2:**
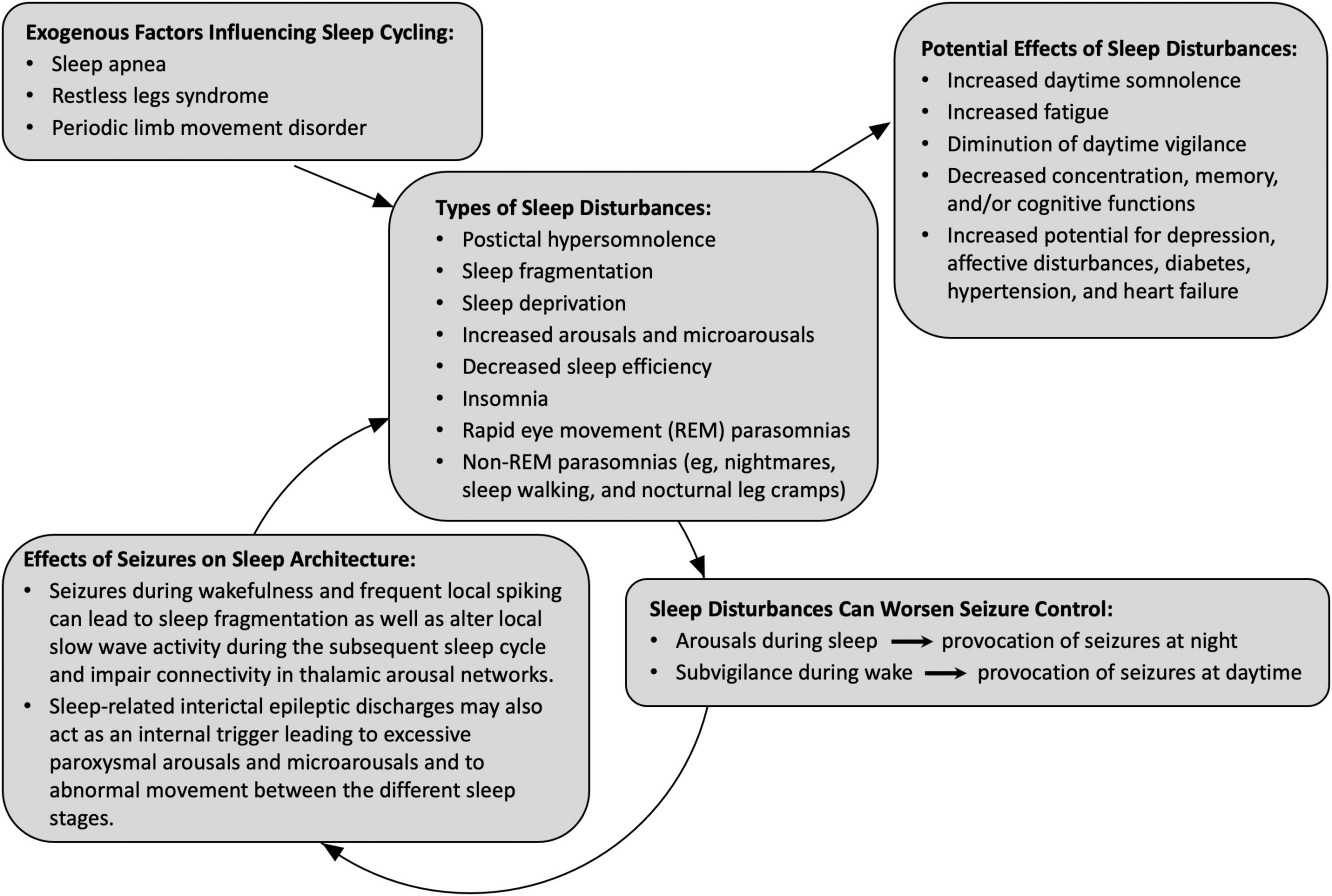
Epilepsy and sleep fragmentation forms a vicious cycle. In people with epilepsy, postictal hypersomnolence, sleep fragmentation, sleep deprivation, increased arousals, and decreased sleep efficiency can have negative diurnal consequences such as increased daytime somnolence and increased fatigue; thereby, leading to affective disturbances, deficits in neuropsychological (mnestic) and cognitive functions, and impairing social productivity and quality of life. Sleep fragmentation and daytime somnolence can also provoke or facilitate further seizures. An arrow represents a causal association with the cause leading to the effect.

Sleep has a critical restorative function and the complex interplay between epilepsy and sleep–wake disorders constitute a vicious cycle (Figure 2). PwE and their caregivers may attribute consistent fatigue and daytime somnolence to the epileptic disease process or as side effects of anti‐seizure medications (ASMs); however, it is imperative to identify and manage comorbid sleep–wake disorders as sleep disturbances can worsen seizure control.^11^

### 2.1 Complex interplay between epilepsy and sleep–wake disorders

Under healthy circumstances, people cycle through all stages of sleep multiple times per night. Each sleep cycle consists of stage N1 (alpha and more than 50% theta waves, lasting several minutes), stage N2 (predominantly theta activity together with sleep spindles and k‐complexes), stage N3 (slow wave deep sleep consisting of more than 20% delta waves), and the rapid eye movement (REM) sleep stage (which increases with each cycle over the course of the night and ranges from 10 to 60 minutes).

In a study in people with focal temporal lobe epilepsy, seizures during wakefulness had a significant impact on sleep structure during the following night (increased stage N1, decreased stages N2 and N3, increased time to first REM period, and decreased REM sleep) leading to decreased sleep efficiency (defined as the relationship between total time spent asleep and the total amount of time spent in bed) and increased overall drowsiness.^12^ Importantly, interictal epileptic discharges, arousals, and microarousals disrupt sleep architecture and favor the occurrence of non‐epileptic motor events, and potentially exacerbate the intensity and length of sleep‐related seizures (Figure S1).^13^

### 2.2 Diagnosis of sleep–wake disorders in PwE

To improve seizure outcomes, it is imperative for clinicians to construct a detailed clinical history of patients and their family members as summarized in Table 1. This includes an evaluation of sleep habits and schedule, sleep complaints, hypersomnolence, reduced diurnal vigilance, circadian rhythm disorders, and influence of sleep on a patient's seizure severity and frequency, as well as any report of reinforced snoring or apnea, weight gain, frequent limb movements during night time, refractory hypertension, mnestic deficits, impairment of impulse control, and impairment of impulse control or affective stability.^14^, ^15^

**TABLE 1:** Clinical consideration of various factors in diagnosis of sleep–wake disorders in patients with drug‐resistant epilepsy.

Clinical Evaluation	Sleep habits and schedule Sleep complaints Hypersomnolence Reduced diurnal vigilance Circadian rhythm disorders Frequent leg or limb movements during sleep	Influence of sleep on a patient's seizure severity and frequency Reports of reinforced snoring or apnea Weight gain Mnestic deficits Impairment of impulse control Impairment of affective stability
Consideration of Risk Factors Related to Sleep–Wake Disorders	Older age Longer duration of epilepsy Higher body mass index (BMI) History of hypertension Higher score on the Sleep Apnea Scale of the Sleep Disorders Questionnaire	Larger neck circumference Hypertonia Absence of nocturnal dipping of blood pressure Congestive heart failure Therapy with benzodiazepines or opiates Comorbid conditions such as anxiety and depression
Other Important Considerations	Risk factors for obstructive sleep apnea Risk factors for restless legs syndrome/periodic limb movement syndrome Low‐ferritin levels Nephropathic diseases	Rheumatoid arthritis Polyneuropathy Diabetes mellitus Neuroleptic or antidepressant medications
Use of Clinical Diagnostic Scales	Pittsburgh Sleep Quality Index Epworth Sleepiness Scale QOLIE 10 QOLIE 31 Liverpool Adverse Event Profile	International Restless Legs Syndrome Rating Scale Beck Depression Scale Berlin Questionnaire for sleep apnea or the Sleep Apnea Scale of the Sleep Disorders Questionnaire to assess respiratory events during sleep

Abbreviation: QOLIE, Quality of Life in Epilepsy.

Various risk factors related to sleep–wake disorders and obstructive sleep apnea (OSA) should also be considered including older age, longer duration of epilepsy, higher body mass index (BMI), history of hypertension, a higher score on the Sleep Apnea Scale of the Sleep Disorders Questionnaire, larger neck circumference, hypertonia, congestive heart failure, and therapy with benzodiazepines or opiates (Table 1).^16^, ^17^ Comorbid conditions such as anxiety and depression may also increase the risk of sleep wake disorders.^18^ Other important considerations include risk factors for restless legs syndrome/periodic limb movement syndrome, low‐ferritin levels, nephropathic diseases, rheumatoid arthritis, polyneuropathy, diabetes mellitus, and neuroleptic or antidepressant medications.^19^

Diagnostic scales can be used to evaluate the presence of comorbid sleep–wake disorders and their influence on the severity of epilepsy (Table 1). Validated patient‐rated questionnaires include the Pittsburgh Sleep Quality Index, Epworth Sleepiness Scale, Quality of Life in Epilepsy (QOLIE) 10, QOLIE 31, Liverpool Adverse Event Profile, International Restless Legs Syndrome Rating Scale, and the Beck Depression Scale. The Berlin Questionnaire for sleep apnea or the Sleep Apnea Scale of the Sleep Disorders Questionnaire are also relevant diagnostic scales to assess respiratory events during sleep.

Indication of sleep–wake disorders can be further investigated using a sleep diary, outpatient actigraphy, or outpatient polygraphy for screening of OSA. Positive diagnostic results can be corroborated in a neurological sleep laboratory using polysomnography and extended electroencephalogram (EEG) diagnostic, to differentiate sleep‐related events into sleep‐related seizures, non‐REM parasomnias, REM behavior disorder, or other forms of sleep‐time movements, as well as to objectify sleep quality (ie, evaluate arousal frequency and relation/stability of different sleep stages, as defined earlier) and sleep efficiency.^20^, ^21^, ^22^

### 2.3 Patient management and clinical advice

The use of various ASMs, antidepressants, and polypharmacological treatments may potentially disrupt sleep architecture in PwE.^23^ Therefore, management of sleep‐related symptoms in PwE requires an individualized approach with consideration of the side‐effect profile and a patient's primary complaints, age, augmentation risks, and preferences; as well as comorbidities—such as sleep apnea and restless legs syndrome—which can lead to a greater disease burden (Table 2). In general, people with DRE should be evaluated in a timely manner for respective epilepsy surgery as well as neurostimulation, such as deep brain stimulation (DBS) and vagus nerve stimulation (VNS Therapy® system).^24^, ^25^, ^26^

**TABLE 2:** Treatment considerations with antiseizure medications (ASMs), antidepressants, polypharmacological treatment, and neurostimulation in patients with epilepsy experiencing sleep–wake disorders.

Comorbidities	Potential effects of treatments in PwE	
	Negative Effects	Positive Effects
Somnological Comorbidities^14^, ^23^, ^108^	Sleep‐impairing anticonvulsant drugs (such as clobazam, levetiracetam, or higher‐dose lamotrigine at night)	Sleep‐promoting ASMs (such as oxcarbazepine and perampanel)
		ASMs that are not known to affect sleep quality (such as lacosamide and zonisamide)
Restless Legs Syndrome or Periodic Limb Movements^109^, ^110^		Symptom‐reducing medications (such as pregabalin and gabapentin)
		Specific dopamine agonists (such as pramipexole, rotigotine, and ropinirole)
		Iron supplementation for individuals with low‐ferritin levels
Obstructive Sleep Apnea^111^, ^112^	Muscle‐relaxing or apnea‐promoting medications (such as benzodiazepines, barbiturates, and opiates) Supine position during sleep VNS Therapy with output current higher than 1.5 mA	Continuous positive airway pressure (CPAP) Reduction of body weight Oral appliances such as lower jaw protrusion splint Hypoglossal nerve stimulation for better seizure control, if appropriate VNS Therapy with output current at night less than 1.25 mA and/or the ON time at night less than 30 sec (based on preliminary evidence; no controlled trial data available)

#### 2.3.1 Surgical treatment

The option of epilepsy surgery should be evaluated in people with DRE as several potential benefits have been reported, including improvement in subjective measures of sleep quality and objective measures documented with overnight polysomnography.^27^

#### 2.3.2 Treatment with DBS

Deep brain stimulation of the anterior nucleus of the thalamus (ANT) may induce stimulus‐dependent and voltage‐dependent arousals and have negative neuropsychiatric effects on memory and mood.^28^, ^29^, ^30^ To minimize the arousals, a bilevel programmed therapy is recommended with a reduction of stimulation strength during sleep time (eg, to an amplitude setting of 2 V) and maintaining the wake time stimulation to a tolerable efficacious level (eg, at an amplitude setting of 5 V).^28^, ^30^, ^31^

#### 2.3.3 Treatment with adjunctive VNS Therapy

VNS Therapy at low‐stimulation levels may improve sleep quality and reduce daytime somnolence in individuals receiving low‐stimulus intensities of ≤1.5 mA, but may lead to several potential effects at high‐stimulation levels (eg, an output current of >1.25 mA at night), such as dose‐dependent arousals, the onset of sleep apnea, or worsen sleep apnea.^30^, ^32^, ^33^, ^34^, ^35^

To attenuate potential side effects of arousals, sleep fragmentation, and apnea (in individuals at risk of sleep apnea), we utilize the guidelines listed in Table 3 in our epilepsy center that are based on 15 years of experience with VNS Therapy. An example of VNS parameter settings for the wake and sleep times in a patient with suspected or confirmed sleep apnea is provided in Table S2.

**TABLE 3:** Recommended settings for VNS generators in individuals at risk for sleep apnea.

VNS therapy generator	Output current	ON time	Pulse width	Signal frequency
VNS Therapy generators without responsive stimulation (eg, Model 102 and Model 103)	Reduce to less than 1.5 mA	21 or 30 sec	250 μsec	20 Hz
VNS Therapy generators with responsive automatic stimulation AutoStim mode (eg, Model 106 AspireSR® generator)	Reduce output current to less than 1.5 mA	Reduce ON time^a^; 30 sec in AutoStim mode and 21 or 30 sec in normal mode	250 μsec	20 Hz
VNS Therapy Model 1000 generator (SenTiva®)	Reduce output current at night to less than 1.25 mA^b^	Reduce ON time at night; 30 sec in AutoStim mode and 21 or 30 sec in normal mode (based on preliminary evidence; no controlled trial data available)	250 μsec	20 Hz

^a^ Reduction of output current and ON Time are important considerations as automatic stimulation settings (output current and ON time) higher than the basic stimulation settings can lead to arousals, heavy snoring, and hypopneas inducing a physiological acceleration of cardiac frequency which may be mistaken by the VNS device as a seizure and subsequently lead to a tachycardia triggered “AutoStim.” In the case of high‐output current or long ON Time, this may subsequently induce longer and stronger apnea potentially leading to a vicious respiratory circle during sleep.

^b^ For VNS Therapy Model 1000 (SenTiva®), a bilevel program setting is recommended for the wake time (with higher output current) versus the sleep time (with reduced output current).

## 3 CARDIOVASCULAR DISEASES AND SUDDEN CARDIAC DEATH IN EPILEPSY

### 3.1 Sudden cardiac death (SCD) and sudden unexpected death in epilepsy (SUDEP)

People with epilepsy have a 2–3‐fold higher risk of premature death compared with the general population and about 15% of the premature deaths are due to sudden cardiac death (SCD) or acute myocardial infarctions.^6^

Sudden cardiac death is defined as either a witnessed natural death, heralded by abrupt loss of consciousness, within an hour of onset of acute cardiovascular symptoms; or an unwitnessed, unexpected death of someone who had been in a stable medical condition (asymptomatic) within the previous 24 hours with no evidence of a non‐cardiac cause and presumably due to a cardiac arrhythmia or hemodynamic catastrophe.^36^

Sudden Unexpected Death in Epilepsy (SUDEP) is another category of sudden death in people with epilepsy that occurs under benign circumstances, in the absence of known structural causes of death (other disease, injury, or drowning), and with or without evidence of a terminal seizure.^37^

It is difficult to provide reliable estimates of SUDEP and SCD incidence rates in PwE as the available epidemiological studies did not assess both entities in the same population. Furthermore, in some cases, SCD and SUDEP can be overlapping entities, for example, in individuals in whom sudden death is due to a lethal primary cardiac arrhythmia recorded at the time of death without known cardiac pathology prior to the event.^38^ However, it is assumed that most SUDEP cases are due to primary central apnea following tonic–clonic seizure, whereas SCD generally involves a different physiological mechanism that is primarily related to the heart.^38^ It is estimated that the incidence of SCD in PwE is about 3‐fold higher than the general population with about 16 000 deaths related to SCD per year in the United States compared with about 3000–4000 deaths per year related to SUDEP.^5^

Based on these numbers, it is imperative to strengthen research efforts to mitigate the risk of SCD in epilepsy. Here, we will present advice on the diagnosis and management of PwE who may be at risk for SCD (or myocardial infarction).

### 3.2 Cardiac complications due to recurrent seizures (‘epileptic heart’)

**FIGURE 3:**
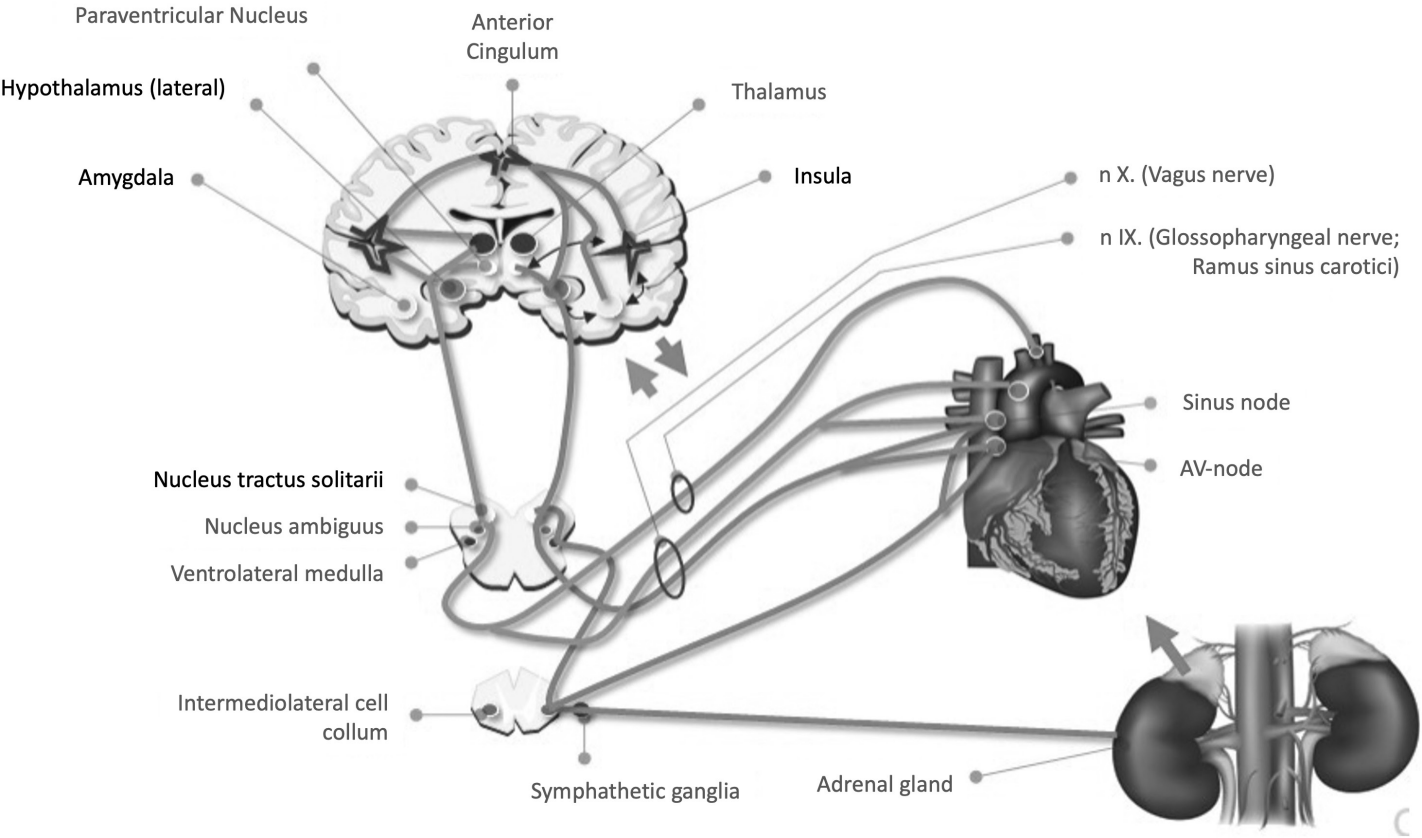
Abnormal neuronal electrical activity during a seizure may lead to autonomic symptoms at the initial stages, during its propagation, or during the postictal phase. Autonomic symptoms include changes in the cardiovascular system, respiratory system, gastrointestinal system, and urinary system, as well as activation of the adrenomedullary hormonal system. Repeated hypoxemia, myocardial ischemia, and catecholaminergic toxicity related to seizures have the potential for cardiomyocyte vacuolization and interstitial cardiac fibrosis.

The epileptic disorder, acute seizure‐related effects, and/or certain ASMs over an extended time can affect autonomic control of the heart and lungs as well as damage the structural integrity of the cardiovascular system (Figure 3). About 60%–80% of PwE report cardiovascular comorbidities with an increased risk of cardiac arrhythmias, ventricular fibrillation, coronary heart disease, angina pectoris, and myocardial infarction.^39^, ^40^, ^41^

Autonomic dysfunction has been linked to an increased risk for SCD regardless of the presence of structural heart disease. The duration of epilepsy with recurrent seizures is likely to be correlated with the incremental development of cardiac damage with myocardial catecholaminergic toxicity and cardiovascular diseases.^6^ Consequently, cardiac fibrosis can develop over time from the repetitive catecholaminergic toxicity of seizures, and this structural change has been identified in about one‐third of PwE from post‐mortem analysis.^5^, ^6^ Based on such observations, the so‐called “epileptic heart” has been proposed by Verrier and colleagues as “a heart and coronary vasculature damaged by chronic epilepsy as a result of repeated surges in catecholamines and hypoxemia leading to electrical and mechanical dysfunction”.^5^ The potential consequences of incremental perivascular and interstitial fibrotic microlesions in the heart may contribute to or cause disturbed conduction of cardiac excitation, heterogeneous cardiac repolarization, and heterotopic excitation; thereby serving as potential sites for the generation and maintenance of fatal arrhythmias such as seizure‐induced ventricular tachycardia or fibrillation.^6^

In PwE, alterations of T‐wave alternans (TWAs) as an indicator of disturbed cardiac repolarization may be of particular interest. TWA is defined as a subtle beat‐to‐beat fluctuation of T‐wave amplitude and morphology of the ST segment or T‐wave.^6^, ^42^ Elevated levels of TWA above 47 μV are associated with an increased risk of lethal arrhythmias, ventricular tachyarrhythmia, and SCD in the cardiac population.^5^ In line with the increased risk of SCD, there is increasing evidence that elevated TWA levels are frequently found in people with chronic epilepsy.^6^, ^42^, ^43^

### 3.3 Acute seizure‐related cardiac complications

#### 3.3.1 Ictal asystole

An ictal asystole is defined as an R–R interval longer than 3 seconds. Ictal asystole is a rare event (affecting about 0.3% of patients with DRE) but can be a serious complication of epileptic seizures leading to syncope and traumatic falls.^44^ Ictal asystole can be difficult to diagnose as cardiac arrhythmia and seizures can lead to similar effects. Ictal asystole can often be identified by sudden loss of muscle tone or the presence of bilateral asymmetric jerky limb movements during a seizure, and electrocardiogram (ECG) monitoring is necessary to confirm diagnosis. Ictal asystole has a high‐recurrence risk which may require cardiac pacemaker implantation if seizure‐freedom cannot be achieved.^44^

#### 3.3.2 Sinus tachycardia

Seizure‐related increases in heart rate and tachycardia (>100 beats per minute) are observed in over 80% of PwE and indicate enhanced sympathetic activity and/or reduced cardiac vagal tone.^45^ It is commonly considered to be a benign condition and may be used for automated seizure detection algorithms.

#### 3.3.3 Ventricular tachycardia

Seizure‐related interictal cardiac complications include altered cardiac autonomic function with decreased heart rate variability and abnormalities of cardiac repolarization features, possibly facilitating the onset of ventricular tachyarrhythmias.^6^ Seizure‐related ventricular tachycardia is a rarely reported event, and when reported, it is related to generalized or focal to bilateral tonic–clonic seizures (TCS).^6^

#### 3.3.4 Takotsubo cardiomyopathy and myocardial infarction

Seizure‐related myocardial infarction is rare and predominantly occurs with TCS in elderly people or in those with coronary artery disease.^6^ Asymptomatic increases of cardiac injury markers in association with epileptic seizures can be interpreted as a mild form of neurogenic stress (Takotsubo) cardiomyopathy.^6^ Takotsubo cardiomyopathy is linked to high‐catecholamine levels that induce cardiac contraction band necrosis with symptoms of chest pain, elevated troponin levels, transient ventricular motion abnormalities, and ECG alterations, similar to those of acute myocardial infarction in the absence of coronary artery disease. The symptoms are commonly reversible, and the prognosis is mostly favorable. In PwE, Takotsubo cardiomyopathy has been reported predominantly in association with TCS or status epilepticus.^6^, ^46^

### 3.4 Cardiac effects of ASMs

ASMs are sometimes described as an independent risk factor for SCD; however, ASMs usually do not exert clinically relevant effects on cardiac repolarization properties (eg, QT intervals) but may worsen pre‐existing conduction abnormalities even at regularly prescribed daily dose levels.^6^, ^39^, ^47^ Importantly, sodium channel‐blocking agents (eg, carbamazepine, lacosamide, lamotrigine, oxcarbazepine, and phenytoin) at high doses or in combination with other sodium channel blocking ASMs were reported to cause cardiac arrhythmias even in the absence of pre‐existing cardiac diseases.^6^ Furthermore, enzyme‐inducing ASMs (eg, carbamazepine, phenobarbital, phenytoin, and primidone) worsen the lipid profile and other serological markers of vascular risk via induction of the hepatic cytochrome P450 system; thereby, facilitating accelerated atherosclerosis.^48^, ^49^

### 3.5 Diagnosis of cardiovascular comorbidities

Starting from epilepsy diagnosis, patients should be evaluated for cardiovascular comorbidities associated with SCD risk and cardiac arrhythmias. It is important to identify if the epileptic disorder, acute seizure activity, and/or ASMs are responsible for potential cardiac findings that may increase the risk of SCD.

The clinical and medication history should be assessed for risk factors, for example, congenital heart disease, arrhythmia symptoms, ventricular dysfunction, dyspnea, chest pain, edema, emotional stress, hypertension, hyperlipidemia, diabetes mellitus, hyperlipidemia, and smoking, as well as any family history of SCD or cardiovascular pathologies.^36^

A 12‐lead ECG is advisable at first epilepsy diagnosis and with new onset of palpitations, episodes with loss of consciousness in the absence of typical seizure symptoms, or other cardiac complaints, as well as in patients upon polypharmacotherapy including two or more sodium channel blocking agents, antihypertensive or antiarrhythmic drugs, or other drugs potentially targeting the heart (such as neuroleptics and antidepressants). Continuous video‐EEG monitoring can be used to record seizure‐related arrhythmias incidentally or shortly after a TCS.^6^ In patients with new onset syncope or episodes of transient loss of consciousness, 12‐lead ECG, Holter recordings for 24–72 hours, or ECG patches can be used to detect epilepsy‐related cardiac arrhythmias.^6^ PwE at risk for cardiac complications or experiencing new onset of chest pain after seizures, should be referred to a cardiologist for further evaluation.

### 3.6 Patient management and clinical advice

If complete seizure control is not achievable in individuals who experience seizure‐related syncope or falls, we advise clinicians to consider implantation of a cardiac pacemaker for ictal bradyarrhythmia or a defibrillator for ictal/postictal ventricular tachycardia or fibrillation.^6^

For individuals who are suspected of peri‐ictal atrial fibrillation and who do not achieve complete seizure control, we advise a 12‐lead ECG, Holter recordings for 24–72 hours, or loop recorder, as well as consideration of rhythm control and referral to a cardiologist.^6^ The benefit of anticoagulation must be weighed against the risk of hemorrhage following seizure‐related falls and injuries.

Enzyme‐inducing ASMs should be avoided at least in individuals with a cardiovascular risk profile and a switch to modern drugs should be considered if possible.^6^

Caution is also warranted with high doses of sodium channel blockers and combinations of such therapies should be avoided as it can lead to potentially life‐threatening cardiac arrhythmia.^50^, ^51^ We would advise reconsidering the ASM strategy or the antihypertensive treatment, especially in people receiving concomitant antihypertensive or antiarrhythmic drugs. A 12‐lead ECG assessment before and about 2 weeks after ASM change is advised to exclude sinus node dysfunction, AV block, or ventricular tachyarrhythmias.^6^

#### 3.6.1 Vagus nerve stimulation and beneficial effects on T‐wave alternans

Reduced TWA levels, increased heart rate variability, improved baroreflex sensitivity, and fewer episodes of ventricular tachycardia are evident following adjunctive VNS Therapy, suggesting that VNS Therapy has potential cardioprotective effects and can considerably reverse or compensate epilepsy‐related cardiac dysfunction.^6^, ^42^, ^52^ VNS Therapy may provide protection in PwE who are at increased risk of cardiac disease by reducing seizure frequency and suppressing cardiac electrical instability.^5^ In individuals receiving VNS Therapy, very rare episodic bradycardia or asystole due to VNS should be considered if the onset of new symptoms or loss of consciousness in the absence of typical seizures is reported.^6^

## 4 COGNITIVE DYSFUNCTION IN EPILEPSY

A long duration of epilepsy and treatment with ASM polypharmacy correlates with a devastating impact on cognitive function and has a profound influence on a person's daily functioning and quality of life. Cognitive impairments are sometimes considered to be secondary to epilepsy or to be caused by epilepsy. However, some form of cognitive deficit is commonly present in about 70% of people with newly diagnosed epilepsy and new‐onset epilepsy, and cognitive deficits have also been shown to exist in some patients prior to the onset of epilepsy.^7^, ^8^ The current understanding is that there is a strong connection between epileptic dysfunction and cognitive impairments, either due to a common underlying pathological process or they may be related in a bidirectional way.^53^ Overall, the cognitive decline appears to result from synergistic effects between initial and acquired lesions as well as aging rather than from convulsive seizures progressively altering the brain structure.

### 4.1 Diagnosis of cognitive impairments in PwE

To evaluate a patient's cognitive and psychological functions, a detailed clinical history should be constructed about daily functioning, the decline in intellectual performance and executive function, learning disabilities, language dysfunction, memory dysfunction, and attention deficits with home maintenance, school, work, or recreational activities. In addition, a neuropsychological evaluation should be performed at least when there are signs or symptoms of a focal cognitive impairment or when evaluating the effects of the disorder and its treatment.^54^ Possible presence of secondary hippocampal sclerosis should be assessed using MRI.

**FIGURE 4:**
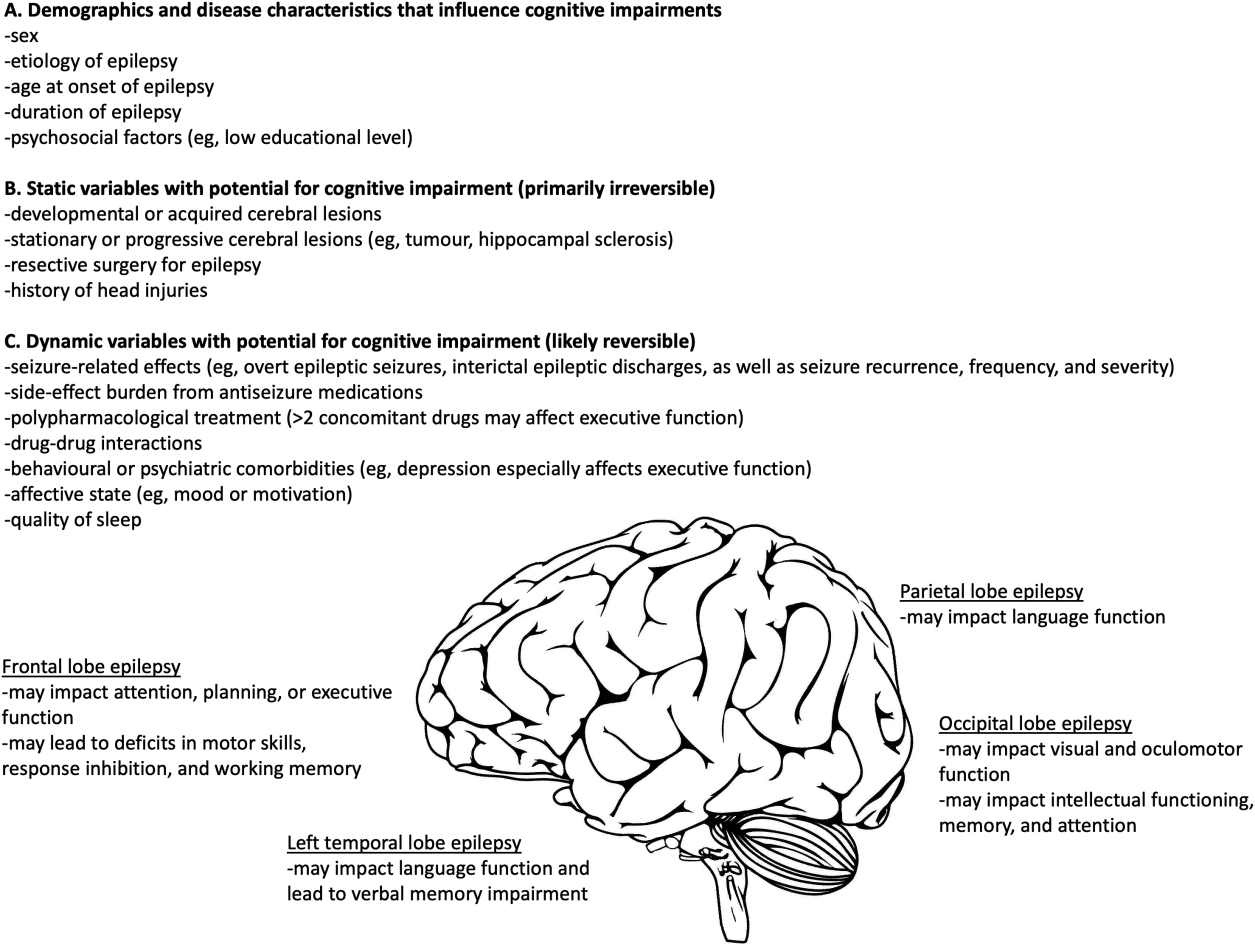
The etiology of cognitive deficits in people with epilepsy is often multi‐factorial. (A) Certain demographic and disease characteristics have been shown to be associated with higher levels of neurocognitive impairments, such as early age of epilepsy onset. (B) Static variables with potential for cognitive impairment are primarily irreversible, such as static or progressive neurological structural alterations and epilepsy surgery. (C) Dynamic variables that influence cognitive impairments in epilepsy are likely reversible, such as seizure frequency and severity; however, abnormal excessive neural activity can permanently modify the synaptic organization and lead to neuronal loss and metabolic dysfunction. In some patients, the site of a focal lesion can lead to specific cognitive effects, for example, parietal lobe epilepsy may impact language function.

As listed in Figure 4, the demographics, disease characteristics (such as early age of epilepsy onset and duration of epilepsy), underlying neuropathologies, as well as static and dynamic variables with potential for cognitive impairment, should be evaluated.^55^, ^56^ While some factors (eg, location and severity of underlying lesions) are static and cause chronic impairments; other factors (eg, seizure frequency and severity) are dynamic and potentially reversible, and may be manageable with clinical intervention.^53^, ^56^, ^57^

### 4.2 Patient management and clinical advice

A patient's cognitive, physical, social, emotional, recreational, executive, and vocational functioning should be considered at all stages of epilepsy management with a periodic longitudinal assessment based on subjective questionnaires and short screening tests. Early detection and subsequent treatment may prevent detrimental effects on cognitive and educational development.

#### 4.2.1 Effects of ASMs on cognition

As some ASMs are known to impact cognitive function, a patient's cognitive status should be reviewed at epilepsy onset, prior to the initial treatment, and over the course of treatment. This process will allow monitoring of the epileptic disorder and to qualify if subsequent changes are related to the treatment or the underlying pathology. In general, treatments with ASMs such as topiramate and zonisamide should be avoided.^57^, ^58^, ^59^, ^60^ In addition, treatment with more than two ASMs should be avoided as the risk for deficits in executive function increases with each additional ASM in combination therapy.^61^

An EpiTrack evaluation is recommended in the context of therapeutic interventions. EpiTrack is a brief (15‐minute) screening tool that can be performed by neuropsychologists or trained clinicians (including trained epilepsy nurses) for objective detection and tracking of cognitive side effects of ASMs, such as word retrieval difficulties (dysnomia), attention, and executive function.^53^, ^62^ Performance on the EpiTrack can also be used to assess other dynamic factors related to cognition, such as seizure frequency and severity, VNS or DBS therapy, and depression.^59^

#### 4.2.2 Neurostimulation and surgical considerations

Polypharmacy is known to have a negative impact on executive functioning, and we recommend that patients with DRE be evaluated in a timely manner for resective epilepsy surgery followed by evaluation for neurostimulation procedures. Such treatments may have more pronounced clinical effects in improving not only seizure outcomes but also cognitive and neuropsychological outcomes, especially with earlier intervention.^63^, ^64^ Specifically, patients who have higher scores in executive function and attention parameters may have more stable neural connectivity which may be a prognostic marker for epilepsy surgery or neurostimulation.^53^, ^65^

Certain epilepsy surgeries (such as hemispheric neurosurgery and resective surgery for temporal lobe or extra‐temporal lobe epilepsies) have been followed by an initial short‐term loss in cognitive level and memory due to the surgical procedures, but patients usually exhibit long‐term stable improvement in areas such as verbal memory and neuropsychological parameters that may be likely due to seizure control and a reduced ASM load.^53^, ^66^

#### 4.2.3 Treatment with DBS

Patients receiving DBS of the ANT were more likely to report depression or memory problems as adverse events.^67^ However, the long‐term findings from the SANTE study did not reveal any significant cognitive or neurobehavioral decline over time, and improvements were noted in total recall, immediate visual recall, design fluency, problem‐solving, and simple visual attention.^29^

#### 4.2.4 Treatment with adjunctive VNS Therapy

The overall effect of VNS Therapy on cognition remains unclear.^63^ The available data suggests that adjunctive VNS treatment has immediate improvement in working memory performance and may have long‐term benefits in word retention, short‐term memory, concentration, verbal communication, and progress in schoolwork following 1–2 years post‐implantation.^68^ In a prospective study, a significant enhancement in verbal memory performance was observed after 6 weeks of VNS treatment.^69^ Notably, the improvement in cognitive outcomes may benefit from an earlier intervention with VNS Therapy.^70^

Studies have shown that VNS Therapy has the potential to reduce interictal epileptic discharges (IEDs) in patients with DRE.^71^, ^72^ IEDs are spikes or spike–wave complexes that occur outside the seizure onset zone and may impair memory encoding, word retrieval, and cognition.^73^ When cyclic VNS was turned ON, subjects made fewer errors in a working memory performance task indicating that VNS Therapy improves working memory performance and enhances visual attention in patients with DRE.^74^

## 5 DEPRESSION IN EPILEPSY

About 30% of patients with controlled epilepsy and about 60% of patients with DRE report depressive symptoms and poor health‐related quality of life.^9^ Depression in epilepsy may lead to worse seizure outcome, significant functional and psychosocial disability, a higher rate of self‐injurious behavior, and an increased risk of suicidal ideation and attempt.^75^, ^76^

Suicide is one of the foremost external causes of death in epilepsy patients and the highest risk of suicide occurs during the first 6 months after epilepsy diagnosis and is especially high in patients with a history of comorbid psychiatric disease.^77^, ^78^ Although depression is a common and serious comorbidity, it is often not recognized or adequately treated in PwE as the symptoms can fluctuate over time and may be interpreted as a consequence of the disease process or side effects of ASMs.^9^

### 5.1 Epilepsy and depression have a complex bidirectional relationship

Comorbid depression and seizure propensity have several shared risk factors including low‐education level, unemployment, compliance issues with ASMs, polypharmacological treatment, anxiety, and stigma due to disease symptoms and social isolation.^79^ Other bidirectional associations between epilepsy and depression have been noted: depression raises the risk of epilepsy onset, depression is a significant predictor of refractory seizure, and incident epilepsy risk is proportional to depression severity.^80^, ^81^ In a longitudinal cohort study, PwE (versus matched controls) were found to have an increased onset of depression, anxiety, and suicidality up to 3 years prior to diagnosis of epilepsy as well as after epilepsy diagnosis.^82^ Cumulatively, these various relationships suggest that there are common underlying pathophysiological mechanisms for a lower seizure threshold and an increased risk for psychiatric disorders and suicide.^83^

### 5.2 Screening for depression in epilepsy

A comprehensive evaluation should be conducted of seizure severity and frequency, recent changes in ASMs or anti‐depressive treatment, number and symptoms of depressive episodes, and family history of psychiatric illness (particularly depression).

Depression as a comorbidity of epilepsy may present with sub‐syndromic depressive episodes (such as interictal dysphoric disorder) and clinicians should be aware of differential diagnosis, such as anxiety, psychosis, aggression, bipolar depression, drug–drug interactions, potential negative psychotropic effects of ASMs, and signs of depression following epilepsy surgery.^84^, ^85^ Adverse events related to ASMs and antidepressants should be assessed to identify overlaps.

Diagnosing mood disorders in PwE is challenging and can be further complicated for neurologists depending on their level of training in psychiatry or due to time constraints in daily clinical practice. Validated screening tools can increase the detection of major depression, anxiety, and signs of suicidality in previously undiagnosed patients, even in a busy outpatient clinic.^86^

We recommend asking patients to complete the following tools at each visit prior to seeing the physician, for example, while they are in the waiting room. The 6‐item Neurological Disorders Depression Inventory for Epilepsy (NDDI‐E) allows rapid objective screening of depressive episodes and suicide risk, while distinguishing neurocognitive deficits and possible adverse effects of ASMs.^87^ The Emotional Thermometer (ET) is a simple screening tool to detect and monitor emotional disorders.^88^ The 21‐item Liverpool Adverse Events Profile (LAEP) can be used to systematically measure adverse effects of ASMs.^89^ In general, NDDI‐E and ET have a high negative predictive value but a lower positive predictive value.^90^ The low scores indicate a negative assessment and are highly reliable. A score higher than the cut‐off scores may potentially indicate a positive assessment of depression but necessitates further clinical assessment for confirmation.

Additional useful screening tools include the Patient Health Questionnaire‐9 (PHQ‐9), Hospital Anxiety and Depression Scale (HADS), and Beck Depression Inventory‐II (BDI‐II).

Importantly, the final judgment on suicidality risk should depend on clinical assessment and should not be based on the results from the screening tools. Patients who are suspected of potential moderate to major depressive symptoms or as having a suicide risk, should be referred to a psychiatrist or neuropsychiatrist for further evaluation.

### 5.3 Patient management and clinical advice

#### 5.3.1 Nonpharmacologic and pharmacologic antidepressant treatment in PwE

Patients diagnosed with “mild” depression may benefit from pharmacologic therapy (selective serotonin reuptake inhibitors [SSRIs] are recommended as a first‐line treatment) and supplemental nonpharmacologic supportive therapy (such as interpersonal psychotherapy, exercise therapy, and cognitive‐behavioral therapy).^91^

Patients with “moderate or major” depression may also benefit from first‐line treatment with SSRIs and nonpharmacologic supportive therapy.^91^ The antidepressants may potentially reduce seizure frequency and lead to an improvement in mood.^92^ However, in the case of nonresponse or only partial respondence, compliance to medication should be assessed prior to switching to alternative drugs (such as venlafaxine) or adding mirtazapine to SSRI.^91^ Further studies have shown that treatment with SSRIs are not associated with increased seizure rates in people with and without epilepsy (compared with placebo).^93^, ^94^

Notably, caution is advised with moclobemide, clomipramine, maprotiline, and bupropion, as these drugs may increase seizure risk; and especially at a higher dose range for the last three listed drugs.^93^

Antidepressants should be maintained for a duration of at least 6–9 months, depending on response and previous history of depressive episodes, and should be reduced stepwise, as appropriate.^91^

Most antidepressants inhibit one or more CYP450 isoenzymes and are metabolized in the liver; for example, inhibition of P450 by SSRIs can result in the accumulation of ASMs in some cases or may lead to toxic serotonin syndrome. Some ASMs are potent CYP450 enzyme inducers and can accelerate the metabolism of antidepressants. An alert was issued by the US FDA in 2008 raising concerns that ASMs may increase the risk of suicidal thoughts and behavior.^95^ This remains a controversial topic as an ASM may have negative or positive psychotropic properties (Table 4).^96^

**TABLE 4:** Psychotropic effects of antiseizure medications. Adopted from Perucca et al, 2013.^96^

ASMs	Psychotropic effects	
	Negative	Positive
First generation		
Barbiturates	Depression *Children and individuals with intellectual disabilities*: hyperactivity, irritability, and aggression	–
Phenytoin	Psychosis (particularly at high‐serum levels)	Possible mood‐stabilizing effects?
Ethosuximide	Psychosis	–
Benzodiazepines	*Children, elderly, and individuals with intellectual disabilities*: hyperactivity, irritability, and aggression	Anxiolytic effects
Carbamazepine	–	Mood‐stabilizing effects
Valproate	–	Mood‐stabilizing effects
Second generation		
Vigabatrin	Psychosis, depression *Children*: hyperactivity, aggression, and agitation	–
Zonisamide	Psychosis, depression, and irritability	–
Lamotrigine	*Individuals with intellectual disabilities*: hyperactivity, irritability, and aggression	Mood‐stabilizing effects Possible mild antidepressant effects?
Felbamate	Anxiety, psychosis	–
Gabapentin	*Children and individuals with intellectual disabilities*: hyperactivity, aggression, and irritability	Anxiolytic effects
Topiramate	Depression, psychosis, and irritability	Anti‐impulsive effects Possible antidepressant effects?
Tiagabine	Irritability	–
Oxcarbazepine	–	Possible mood‐stabilizing effects?
Levetiracetam	Irritability, aggression, depression, and psychosis	–
Stiripentol	Hyperactivity, irritability, and aggression	–
Pregabalin	Depression	Anxiolytic effects
Third generation		
Rufinamide	–	–
Lacosamide	–	–
Eslicarbazepine acetate	–	–
Retigabine	–	–

Caution should be applied when ASMs are changed in female patients in preparation for pregnancy. For example, changing carbamazepine or another ASM that has teratogenic potency to levetiracetam includes the risk of developing subsequent depression.^97^

We advise clinicians to be vigilant and screen for suicide, but not delay treatment with ASM and/or antidepressants if clinically indicated. We also recommend psychiatric consultation prior to the initiation of psychotherapeutic therapies, as appropriate.^91^

#### 5.3.2 Resective epilepsy surgery and risk for depression

Patients receiving epilepsy surgery are at risk of developing major depression, which is more prominent if the mesial temporal lobe is resected.^98^ Therefore, patients experiencing a major depressive episode should be stabilized prior to epilepsy surgery. Although a pre‐operative history of depression is a risk factor, 13% of post‐operative depressive episodes are de novo. A prediction model for postsurgical depression in temporal lobe surgery has identified 6 predictors including psychiatric history, resection side, relationship status, verbal fluency, age at surgery or testing, and malformations of cortical development on MRI.^99^ Depression may occur within 6–12 months after epilepsy surgery, and careful screening and assessment for depression should be included during pre‐ and postsurgical consultation.

#### 5.3.3 VNS therapy in patients with DRE experiencing comorbid major depression

Patients with DRE who are not surgical candidates should be evaluated for VNS Therapy, especially if comorbid depression is present. In a study in 59 patients with DRE and comorbid depression, the severity of depressive symptoms improved in all patients following one year of adjunctive VNS Therapy and was strongly correlated with a reduction in seizure frequency.^100^ A recent systematic review confirmed improvement in depression in patients with DRE and VNS treatment, whereas the antidepressant effect was unrelated to seizure response.^101^

#### 5.3.4 Treatment with DBS

Some patients treated with DBS may experience depression.^102^ Therefore, DBS in PwE who are experiencing comorbid uncontrolled depression should be used with caution.

#### 5.3.5 Ketogenic diet in patients with DRE experiencing comorbid depression

A longer‐duration ketogenic diet in PwE may have potential positive psychiatric effects independent of seizure reduction or ketone body production.^103^, ^104^ As GABA (γ‐aminobutyric acid) metabolism is generally reduced in depression, an increase in levels of GABA neurotransmitter with a ketogenic diet may also have a positive treatment effect.^105^

#### 5.3.6 Forced normalization

Psychiatric symptoms sometimes occur following seizure control in patients with DRE who were experiencing high‐frequency seizures. This phenomenon is referred to as “forced normalization” (FN). Although most often these symptoms are of a psychotic nature (86%), FN manifests also as mood disorders (26%) or dissociation (5%).^106^ FN can be triggered by ASMs (mainly levetiracetam and vigabatrin), epilepsy surgery, or vagal nerve stimulation.^93^, ^106^ Treatment, particularly of psychosis, may not respond well to drug therapy but tapering or withdrawal of the triggering ASM leads to complete resolution in the majority of cases.^106^ If FN is triggered by VNS Therapy, decreasing the pulse intensity may improve the psychotic symptoms.^106^, ^107^

#### 5.3.7 Other psychiatric comorbidities

We focused on the comorbidity of depression in PwE as it is the most common psychiatric comorbidity. PwE also experiences other psychiatric comorbidities including personality disorders and psychosis, and the latter can be very challenging to manage. For more information on this topic, see Table S1.

## 6 CONCLUSIONS

Management of epilepsy can be challenging, and it is recommended that PwE meet at least once per year with an epileptologist for reassessment of their clinical symptoms, comorbidities, quality of life, and treatment plan.

Treatment with ASMs or neurostimulation should not impair sleep quality, as poor sleep quality or quantity may worsen seizure control. When such side effects cannot be avoided, therapies should be optimized to ensure that therapy‐induced sleep disturbances do not antagonize the anticonvulsant effects of the therapies.

Patients with active epilepsy have a 5.8‐fold increased risk of SCD compared with a 1.6‐fold increased risk in patients with stable epilepsy, highlighting the need to adequately control epileptic seizure symptoms. Caution is warranted with sodium channel blockers in combination or at high doses. For the management of patients with a cardiovascular risk profile, enzyme‐inducing ASMs should be avoided. VNS appears to have beneficial cardioprotective effects.

As cognitive deficits are underreported by patients and their caregivers, clinicians may consider partnering with a trained neuropsychologist for comprehensive testing of cognitive deficits. This will be especially helpful prior to initiating treatment in patients with new onset epilepsy and patients with DRE, to assist in identifying areas of the brain impacted by seizure activity as well as assess global cognition, working memory, verbal fluency, and processing speed. It is also important that differential diagnosis of cognitive and psychological function (such as depression and anxiety) is identified and addressed.

Neurobehavioral domains related to memory and depression should be routinely monitored and evaluated before surgical procedures or neurostimulation as well as during follow‐up care. An EpiTrack evaluation is recommended as part of the follow‐up after neurostimulation procedures.

Careful assessment of depression and suicidal ideation is warranted in PwE. Having access to liaison psychiatric services as part of a multidisciplinary epilepsy care team may also have beneficial effects. Treating comorbid depression will significantly increase a patient's quality of life and may result in a reduction of seizure frequency; and both factors are of particular importance in DRE. VNS Therapy should be considered as a treatment option in patients with DRE, especially if a comorbidity of depression is present.

### 6.1 Summary

Comorbidities can significantly impact health‐related quality of life, seizure frequency, and severity in PwE, and a holistic approach to evaluation and treatment is important.

## CONFLICT OF INTEREST STATEMENT

Over the last 5 years, JP has participated in clinical trials for Eisai, UCB, and BIAL; received research grants from Eisai, Medtronic, UCB, and LivaNova; received speaker honoraria from LivaNova, Eisai, Medtronic, Orion Pharma, and UCB; received support for travel to congresses from LivaNova, Eisai, Medtronic, and UCB; and participated in advisory boards for Arvelle Therapeutics, Novartis, LivaNova, Eisai, Medtronic, UCB and GW Pharmaceuticals.

Over the last 5 years, RS reports lecture and consultancy fees from Angelini, Arvelle, Bial, Desitin, Eisai, Janssen‐Cilag GmbH, LivaNova, Novartis, Precisis GmbH, UCB Pharma, and UnEEG, as well as research grants from the Boll foundation (Kerpen, Germany), BONFOR research funding (Medical Faculty, University of Bonn, Germany), the Federal Ministry of Education and Research (Germany), the Federal Ministry of Health (Germany), and the Verein zur Förderung der Epilepsieforschung e.V. (Bonn, Germany).

Over the last 5 years, BV has received lecture and consulting fees and travel expenses from Bioprojet, EISAI, JAZZ Pharma, LivaNova, MEDTRONIC, GW, Angelini, Neuraxpharm, and UCB.

TvO reports grants, personal fees, and non‐financial support from Novartis Pharma; personal fees from Roche Pharma, Biogen Idec Austria, LivaNova, Indivior, Austria GmbH, Philips, and Almirall; grants from Grossegger & Drbal GmbH and Merck; personal fees and non‐financial support from g.tec GmbH Austria; grants and non‐financial support from Boehringer‐Ingelheim; personal fees and non‐financial support from UCB Pharma; grants and personal fees from Eisai; and personal fees from Arvelle Therapeutics outside the submitted work; and he is co‐chair of the communication committee of the European Academy of Neurology (EAN), co‐chair of the EAN scientific panel for epilepsy, and president of the Österreichische Gesellschaft für Epileptologie (Austrian ILAE chapter).

## ETHICAL PUBLICATION STATEMENT

We confirm that we have read the Journal's position on issues involved in ethical publication and affirm that this report is consistent with those guidelines.

## Supporting information


Figure S1.

Table S1.
Table S2.
