# Supplementary figures and images for: Expert opinion on diagnosis and management of epilepsy‐associated comorbidities
Source: Epilepsia Open. 2023 Nov 27;9(1):15–32. doi: 10.1002/epi4.12851 (PMC10839328; doi:10.1002/epi4.12851)

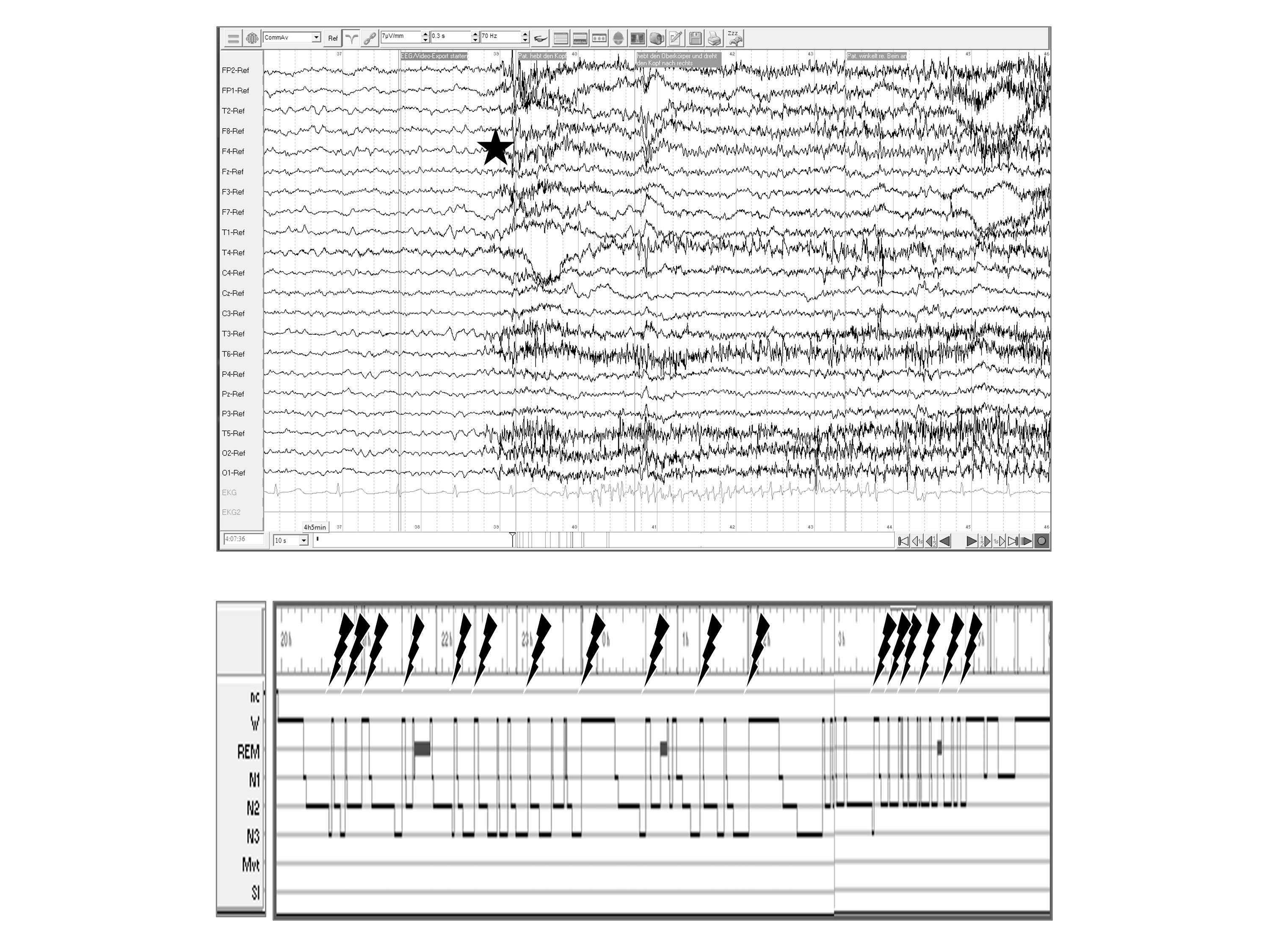

Supplement: Supplementary file 1 — Figure S1. [file EPI4-9-15-s001.tiff]
