# Supplementary material for: Expert opinion on diagnosis and management of epilepsy‐associated comorbidities
Source: Epilepsia Open. 2023 Nov 27;9(1):15–32. doi: 10.1002/epi4.12851 (PMC10839328; doi:10.1002/epi4.12851)
Supplement: Supplementary file 2 — Table S1. Table S2. [file EPI4-9-15-s002.docx]

Supplemental Table S1. References for further reading

| Epilepsy and Sleep Disorders | Voges B, Imbach L. Neurostimulation and sleep in patients with epilepsy—English version. Clin Epileptology. 2023 June. https://link.springer.com/article/10.1007/s10309-023-00600-5.  Nobili L, et al. Standard procedures for the diagnostic pathway of sleep-related epilepsies and comorbid sleep disorders: A European Academy of Neurology, European Sleep Research Society and International League against Epilepsy-Europe consensus review. J Sleep Res. 2020 Dec;29(6):e13184.  McLeod GA et al. Can REM Sleep Localize the Epileptogenic Zone? A Systematic Review and Analysis. Front Neurol. 2020 Jul 24;11:584.  Voges BR, et al. Complex sexual behaviors during sleep as a manifestation of epilepsy: a case series. Sleep. 2019 Mar 1;42(3):zsy233.  Zambrelli E, et al. Laryngeal motility alteration: A missing link between sleep apnea and vagus nerve stimulation for epilepsy. Epilepsia. 2016 Jan;57(1):e24-7.  Halász P. Are Absence Epilepsy and Nocturnal Frontal Lobe Epilepsy System Epilepsies of the Sleep/Wake System? Behav Neurol. 2015;2015:231676.  Voges BR, et al. Deep brain stimulation of anterior nucleus thalami disrupts sleep in epilepsy patients. Epilepsia. 2015 Aug;56(8):e99-e103.  Jain SV, Glauser TA. Effects of epilepsy treatments on sleep architecture and daytime sleepiness: an evidence-based review of objective sleep metrics. Epilepsia. 2014 Jan;55(1):26-37. |
| --- | --- |
| Cardiovascular Dysfunction and Sudden Cardiac Death in Epilepsy | Thijs RD, et al. Autonomic manifestations of epilepsy: emerging pathways to sudden death? Nat Rev Neurol. 2021 Dec;17(12):774-788.  Surges R, et al. Identifying patients with epilepsy at high risk of cardiac death: signs, risk factors and initial management of high risk of cardiac death. Epileptic Disord. 2021 Feb 1;23(1):17-39.  Verrier RL, et al. The Epileptic Heart: Concept and clinical evidence. Epilepsy Behav. 2020 Apr;105:106946.  Shmuely S, et al. The heart of epilepsy: Current views and future concepts. Seizure. 2017 Jan;44:176-183.  van der Lende M, Surges R, Sander JW, Thijs RD. Cardiac arrhythmias during or after epileptic seizures. J Neurol Neurosurg Psychiatry. 2016 Jan;87(1):69-74.  Lamberts RJ, et al. Sudden cardiac arrest in people with epilepsy in the community: Circumstances and risk factors. Neurology. 2015 Jul 21;85(3):212-8. |
| Cognitive Impairments in Epilepsy | Aniwattanapong D, et al. Effect of Vagus Nerve Stimulation on attention and working memory in neuropsychiatric disorders: A Systematic Review. Neuromodulation. 2022 Apr;25(3):343-355.  Lähde N, et al. EpiTrack is a feasible tool for assessing attention and executive functions in patients with refractory epilepsy. Epilepsy Behav. 2021 Feb;115:107691.  Chan AY, et al. Effect of neurostimulation on cognition and mood in refractory epilepsy. Epilepsia Open. 2018 Feb 13;3(1):18-29.  Helmstaedter C, Witt JA. Epilepsy and cognition - A bidirectional relationship? Seizure. 2017 Jul;49:83-89.  Witt JA, Helmstaedter C. Cognition in the early stages of adult epilepsy. Seizure. 2015 Mar;26:65-8.  Witt JA, Helmstaedter C. Monitoring the cognitive effects of antiepileptic pharmacotherapy--approaching the individual patient. Epilepsy Behav. 2013 Mar;26(3):450-6.  Lutz MT, Helmstaedter C. EpiTrack: tracking cognitive side effects of medication on attention and executive functions in patients with epilepsy. Epilepsy Behav. 2005 Dec;7(4):708-14.  Elger CE, et al. Chronic epilepsy and cognition. Lancet Neurol. 2004 Nov;3(11):663-72. |
| Depression in Epilepsy | Mula M. Developments in depression in epilepsy: screening, diagnosis, and treatment. Expert Rev Neurother. 2019 Mar;19(3):269-276.  Calle-Lopez Y, et al. Forced normalization: A systematic review. Epilepsia. 2019;60(8):1610-8.  Mula M. Do anti-epileptic drugs increase suicide in epilepsy? 10 years after the FDA alert. Expert Rev Neurother. 2018 Mar;18(3):177-178.  Josephson CB, Jetté N. Psychiatric comorbidities in epilepsy. Int Rev Psychiatry. 2017 Oct;29(5):409-424.  Agrawal N, et al. Depression correlates with quality of life in people with epilepsy independent of the measures used. Epilepsy Behav. 2016 Sep;62:246-50.  Kanner AM. Management of psychiatric and neurological comorbidities in epilepsy. Nat Rev Neurol. 2016 Feb;12(2):106-16.  Hoppe C, Elger CE. Depression in epilepsy: a critical review from a clinical perspective. Nat Rev Neurol. 2011 Jul 12;7(8):462-72. |

Supplemental Table S2. Example of VNS parameter settings for wake and sleep times in a patient with suspected or confirmed sleep apnea.

| **Wake time** | Basic settings may be 1.75 mA output current, 30 second ON time, 250 µsec pulse width, and 20 Hz frequency.  Autostimulation settings may be 1.75 mA output current, 60 second ON time, 250 µsec pulse width, and 20 Hz frequency. |
| --- | --- |
| **Sleep time** | Basic settings may be reduced to 1.0 or 1.25 mA output current, 21 second ON time, 250 µsec pulse width, 20 Hz frequency.  Autostimulation settings can be adjusted to 1.0 or 1.25 mA output current, 30 second ON time, 250 µsec pulse width, and 20 Hz frequency. |
